# Supplementary material for: Helichrysum stoechas (L.) Moench Inflorescence Extract for Tomato Disease Management
Source: Molecules. 2023 Aug 3;28(15):5861. doi: 10.3390/molecules28155861 (PMC10421166; doi:10.3390/molecules28155861)
Supplement: Supplementary file 1 [file molecules-28-05861-s001.zip › molecules-2509063-supplementary.pdf]

Helichrysum stoechas (L.) Moench Inflorescence Extract for Tomato Diseases Management

E. Sánchez-Hernández, J. Álvarez-Martínez, V. González-García, J. Casanova-Gascón, J. Martín-Gil, and P. Martín-Ramos

SUPPLEMENTARY MATERIALS

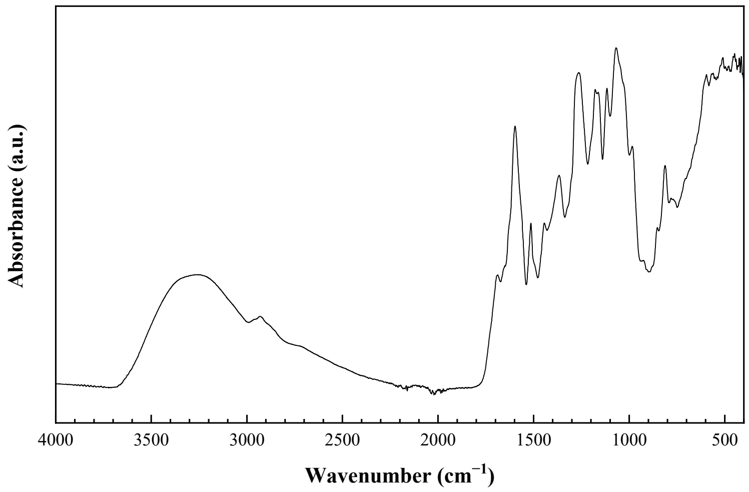

Figure S1. ATR-FTIR spectrum of *Helichrysum stoechas* dried inflorescences.

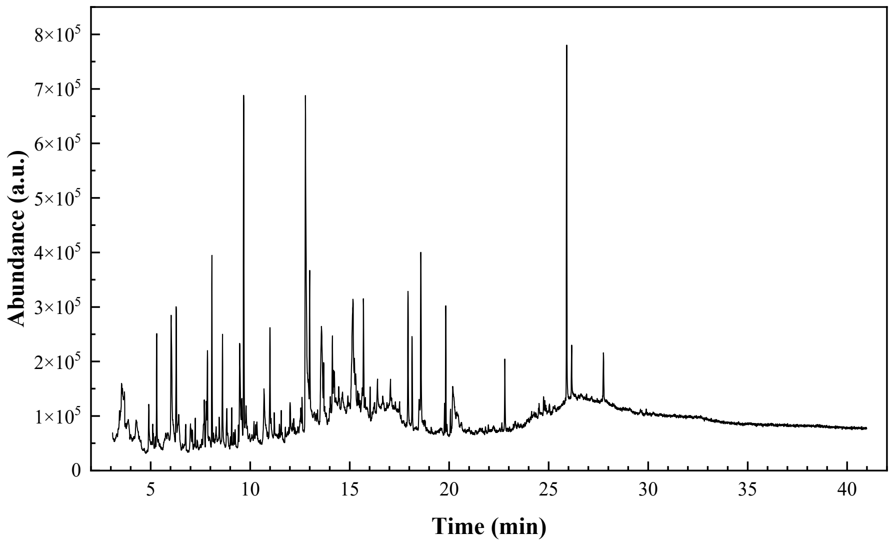

Figure S2. GC-MS chromatogram of *Helichrysum stoechas* hydromethanolic inflorescence extract.

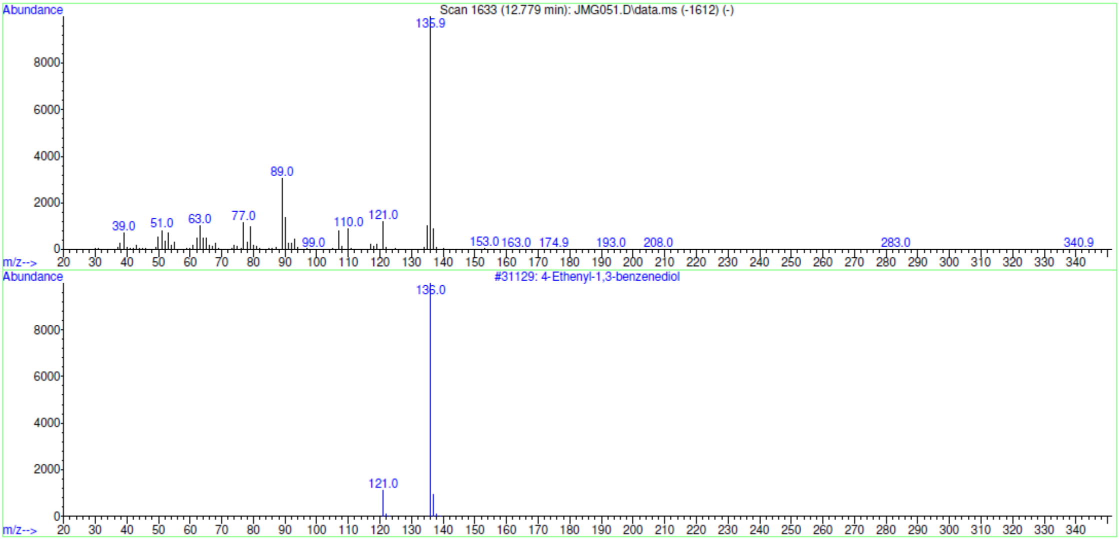

Figure S3. Comparison of MS spectra of 4-ethenyl-1,3-benzenediol with that of the chemical species detected at RT=12.779 min.

Table S1. Efficacies reported in the literature for plant extracts against the six phytopathogenic fungal taxa under study.

Pathogen	Source/Extraction Medium	Plant	Efficacy	Ref.
A. alternata	Water:methanol (1:1 *v/v*)	*Helichrysum stoechas* inflorescences	MIC = 500 μg·mL^{-1}	This work
	Aqueous extracts at 1000 μg·mL^{-1}	*Acacia nilotica* leaves	IR = 35.75%	[1]
		Datura stramonium leaves	IR = 57.50%	
		Allium sativum cloves	IR = 64.75%	
		Zingiber officinale rhizome	IR = 42.25%	
		Amaranthus viridis leaves	IR = 39.25%	
		Azadirachta indica kernel and leaves	IR = 62.75%	
	Aqueous, acetone, ethanol, or methanol extracts at 200,000 μg·mL^{-1}	*Ocinum sanctum* leaves	IR = 44.75%	[2]
		Lantana camara leaves	IR = n.a.–100%	
	Methanol extracts at 7000 μg·mL^{-1}	*Artemisia annua* leaves	IR = 43%	[3]*
		Anadenanthera colubrina barks	IR = 46%	
		Ruta graveolens flowers and leaves	IR = 28%	
	Methanol extracts at 7.5%	*Curcuma longa* leaves	IR = 50%	[4]
		Ocimum tenuiflorum leaves	IR = 51.90%	
		A. indica leaves	IR = 45.71%	
		Aloe vera leaves	IR = 34.28%	
	Aqueous extracts at 15%	*Calotropis gigantea* leaves	IR = 84.88%	[5]
		Alstonia scholaris leaves	IR = 47.66%	
		Z. officinale rhizomes	IR = 65.55%	
		D. stramonium leaves	IR = 68.33%	
		A. sativum cloves	IR = 100%	
		A. indica leaves	IR = 88.22%	
		A. vera leaves	IR = 52.44%	
	Aqueous extract at 4%	*Cinnamomum zeylanicum* bark	n.a.	[6]
	Aqueous extracts at 10%	*Oscimum sanctum*	IR = 18.47%	[7]
		A. indica	IR = 35.83%	
		Eucalyptus globulus	IR = 14.72%	
		A. sativum	IR = 26.38%	
		C. longa	IR = 54.92%	
	Aqueous or methanol extracts at 6%	*Annona muricata* pulp or seeds	IR = 47.9–90.93%	[8]
	Methanol extract	*Phoenix dactylifera* pits	IC_{50} = 2800 μg·mL^{-1}	[9]
	Aqueous (10%) or ethanol extracts (5%)	*Artemisia absinthium*	IR = 40–83.33%	[10]

		Rosmarinus officinalis	IR = 26.67–83.33%	
		D. stramonium	IR = 16.67–83.33%	
		Xanthium strumarium	IR = 30–86.67%	
	Aqueous extract at 20%	*A. indica* leaves	IR = 74.92%	[11]
		Calotropis gigantia leaves	IR = 58.14%	
		C. longa leaves	IR = 66.55%	
		A. sativum cloves	IR = 77.25%	
		Z. officinale rhizomes	IR = 77%	
		Ocimum sanctum leaves	IR = 61.97%	
		Allium cepa bulbs	IR = 74.19%	
		Parthenium hysterophorus leaves	IR = 43.71%	
		Eucalyptus obliqua leaves	IR = 72.82%	
	Aqueous extracts at 10%	*A. indica*	IR = 68.30%	[12]
		Sapindus mukorossi	IR = 73.04%	
		A. sativum	IR = 42.11%	
		Calotropis procera	IR = 20.96%	
		Psidium guajava	IR = 40.93%	
		Vitex negundo	IR = 35.71%	
		Glyricidia maculata	IR = 44.33%	
		Solanum torvum	IR = 31.82%	
		L. camara	IR = 45.33%	
Colletotrichum spp.	Water:methanol (1:1 *v/v*) against *Colletotrichum coccodes*	*H. stoechas* inflorescences	MIC = 375 $\mu g \cdot mL^{-1}$	This work
	Ethanol extracts at 750 $\mu g \cdot mL^{-1}$ against *Colletotrichum acutatum*	*A. sativum* peels	IR ≥ 50%	[13]*
		A. cepa peels	IR ≥ 50%	
	Aqueous extracts at 10% against *Colletotrichum gloeosporioides*	*Ageratum conyzoides* leaves	IR = 56.83%	[14]*
		A. sativum leaves	IR = 100%	
		Jatropha curcas leaves	IR = 49.90%	
		Ocimum basilicum leaves	IR = 100%	
		Vernonia amygdalina leaves	IR = 48.32%	
	Aqueous extracts at 20% against *C. gloeosporioides*	*Achras sapota* leaves or stems	IR = 37.5–42.5%	[15]
		Annona reticulata leaves or stems	IR = 22.5–27.5%	
		Carica papaya leaves or stems	IR = 27.5–35%	
		Citrus lemon leaves or stems	IR = 32.4–100%	
		Dyospiros ebenaster leaves or stems	IR = 35–37.5%	
		Mangifera indica leaves or stems	IR = 25–37.5%	

	Persea americana leaves or stems	IR = 30–100%	
	Pouteria sapota leaves or stems	IR = 12.5–52.5%	
	Spondias purpurea leaves or stems	IR = 37.5–50%	
	Tamarindus indicus leaves or stems	IR = 42.5–65%	
Aqueous extracts at 90% against *Colletotrichum* sp.	*Piper nigrum* seeds	IR = 82.19%	[16]
	Z. officinale rhizomes	IR = 76.98%	
	A. indica leaves	IR = 57.30%	
	C. papaya leaves	IR = 51.97%	
	Nicotiana tabacum leaves	IR = 61.82%	
Methanol extracts at 2000 $\mu g \cdot mL^{-1}$ against *C. gloeosporioides*	*Acacia pennatula* roots	IR = 54.4%	[17]
	Petiveria alliacea leaves	IR = 6.2%	
	Piscidia piscipula leaves	IR = 56.2%	
	Pithecellobium albicans leaves or roots	IR = 4.4–23.1%	
	Pithecellobium dulce leaves or roots	IR = 21.2–81.2%	
	Tribulus cistoides leaves or roots	IR = 23.1%	
	Viguiera dentata leaves	IR = 43.7%	
Aqueous extracts at 20% against *Colletotrichum dematium*	*A. sativum* leaves	IR = 72.73%	[18]
	Z. officinale leaves	IR = 39.83%	
	A. cepa leaves	IR = 53.47%	
	A. indica leaves	IR = 32.47%	
Acetone, aqueous or methanol extracts at 20% against *C. gloeosporioides*	*Prosopis hysterophorus* leaves	IR = 79.6–100%	[19]
	L. camara leaves	IR = 65.2–86.3%	
	Mimosa diplotricha leaves	IR = 26.6–56.7%	
	P. hysterophorus leaves	IR = 28.1–64.8%	
	Echhornia crassipes leaves	IR = 45.2–71.1%	
Methanol extracts at 400,000 $\mu g \cdot mL^{-1}$ against *C. gloeosporioides*	*Bromelia pinguin* pulp or peels	IR < 5%	[20]
	Psidium sartorianum pulp or seeds	IR = 20.4–25.7%	
	S. purpurea Pulp or peels	IR < 5%	
	Echeveria craigiana leaves	IR < 5%	
	Echeveria kimnachii leaves	IR = 23.1%	
	Echeveria subrigida leaves	IR < 5%	
	*P. dulce p*ulp, peles or seeds	IR < 5–21.5%	
	Randia echinocarpa pulp or seeds	IR = 4.73–60.8%	
	Vitex mollis pulp or seeds	IR = 21.4–100%	
	A. sativum bulbs	MIC = n.a.–6250 $\mu g \cdot mL^{-1}$	[21]

	Acetone, aqueous or ethyl acetate extracts against *Colletotrichum lindemuthianum* and *C. dematium*	*Agapanthus caulescens* whole plant	MIC = n.a.–6250 μg·mL^{-1}	
		Syzygium cordatum fruits	MIC = 780–6250 μg·mL^{-1}	
		C. papaya leaves	MIC = n.a.–1560 μg·mL^{-1}	
		Chlrophytym comosum whole plant	MIC = n.a.–12,500 μg·mL^{-1}	
		Ipomoea batatas leaves	n.a.	
	Acetone or ethanol extracts at 5000 μg·mL^{-1} against *C. gloeosporioides*	*Lantana hirta* leaves or flowers	IR = n.a.–100%	[22]
		Argemone ochroleuca leaves-fruits or roots	IR = n.a.–100%	
		Adenophyllum porophyllum leaves	IR = 10–100%	
	Aqueous extract at 70% against *C. gloeosporioides*	*Swietenia macrophylla* seeds	IR = 26.24%	[23]
		Z. officinale rhizomes	IR = 34.87%	
		N. tabacum leaves	IR = 18.54%	
		C. gigantea leaves	IR = 24.38%	
		A. sativum cloves	IR = 60.45%	
		Sonneratia apetala pulp or seeds	IR = 29.64–40.39%	
	Ethanol extract against *Colletotrichum* spp.	*Pimenta dioica* leaves	MIC = 5000–10,000 μg·mL^{-1}	[24]
		Piper auritum leaves	MIC = 40,000 μg·mL^{-1}	
		Plectranthus amboinicus leaves	MIC = 5000–10,000 μg·mL^{-1}	
		P. guajava leaves	MIC > 40,000 μg·mL^{-1}	
	Ethanol extracts at 10,000 μg·mL^{-1} against *C. gloeosporioides*	*Hydnocarpus anthelmintica* fruits	IR = 51.11%	[25]
		Crateva magna fruits	IR = 52.22%	
		Caesalpinia sappan barks	IR = 34.78%	
		Xanthophyllum lanceatum fruits	IR = 20%	
		Carallia brachiata barks	IR = 18.11%	
	Chloroform, hexane, or methanol extracts at 400 μg·mL^{-1} against *Colletotrichum musae*	*Terminalia arjuna* leaves	IR = 35.19–49.63%	[26]
		Clerodendrum infortunatum leaves	IR = 9.63–78.15%	
		Anethum graveolens seeds	IR = 30.74–49.67%	
		Z. officinale rhizomes	IR = 9.26–87.41%	
		A. sativum bulbs	IR = 19.63–34.48%	
		L. camara leaves	IR = 38.15–51.48%	
		N. tabacum leaves	IR = 15.19–41.48%	
		Polygonum hydropiper leaves	IR = 15.19–38.30%	
		Pongamia pinnata seeds	IR = 11.48–37.89%	
		Polyalthia longifolia leaves	IR = 10.37–84.07%	
		Tectona grandis leaves	IR = 27.78–60.59%	
	Aqueous crude extract against *C. acutatum*	*Rhus coriaria* fruits	MIC = 5 μg·mL^{-1}	[27]

	Water:methanol (1:1 *v/v*)	*H. stoechas* inflorescences	MIC = 500 µg·mL^{-1}	This work
	Methanol/water extract (1:1 *v/v*)	*Armeria maritima* flowers	MIC = 750 µg·mL^{-1}	[28]
	Aqueous extracts at 4%	*C. zeylanicum* barks	IR ≤ 30%	[6]
		A. indica leaves	n.a.	
	Aqueous extracts at 5, 10, and 20%	*P. hysterophorus* leaves and flowers	IR = 2.6–15.9%	[29]
		Momordica charantia leaves	IR = 14.4–24.4%	
		A. sativum cloves	IR = 52.6–63.3%	
		E. globulus leaves	IR = 34.3–61.8%	
		C. procera leaves	n.a.	
		A. vera leaves	IR = 16.6%	
		Beta vulgaris roots	IR = 6.3–10.3%	
		D. stramonium leaves	IR = 61.1%	
	Aqueous or propanol extracts at 1%	*Punica granatum* fruits	IR = 62–78%	[30]
	Chloroform, ethyl acetate, methanol or hexane extracts	*Cestrum nocturnum* flowers	MIC = 500–1000 µg·mL^{-1}	[31]
F. oxysporum	Crude extracts at 5, 10, and 20%	*A. indica* leaves	IR = 24.1–62.0%	[32]
		O. sanctum leaves	IR = 7.0–17.0%	
		Datura metel leaves	IR = 10.1–34.2%	
		Cassia alata leaves	IR = 46.8–74.7%	
		Asparagus racemosus roots	IR = 44.3–57.0%	
		A. sativum bulbs	IR = 17.6–34.2%	
		Z. officinale tubers	IR = 23.7–39.5%	
	Ethanol extracts	*Flourensia microphylla* leaves	MIC = 1500 µL·L^{-1}	[33]
		Flourensia cernua leaves	MIC = 1500 µL·L^{-1}	
		Flourensia retinophylla leaves	MIC = 1500 µL·L^{-1}	
	Aqueous extracts at 5–50%	*Moringa oleifera* leaves, roots or pud coats	IR = 36–100%	[34]
	Aqueous extracts at 25%	*A. nilotica* leaves	IR = 82%	[35]
		A. zapota leaves	IR = 34.8%	
		D. stramonium leaves	IR = 67.5%	
		E. officinalis leaves	IR = 79.5%	
		E. globulus leaves	IR = 59.3%	
		L. inermis leaves	IR = 82.0%	
		M. elengi leaves	IR = 86.0%	
		P. pterocarpum leaves	IR = 53.3%	
		P. longifolia leaves	IR = 36.3%	
		P. juliflora leaves	IR = 80.3%	

		P. granatum leaves	IR = 73.8%	
		Syzygium. cumini leaves	IR = 69.5%	
	Aqueous extracts at 0.2% (*w/v*)	*Filipendula* spp. flowers	IR = 95.9%	[36]
		A. sativum	IR = 81.4%	
	Ethanolic extract	*Mentha spicata* leaves and stems	MIC = 5%	[37]
	Aqueous extract	*A. sativum* leaves	MIC = 7000 $\mu g \cdot mL^{-1}$	[38]
V. dahliae	Water:methanol (1:1 *v/v*)	*H. stoechas* inflorescences	MIC = 375 $\mu g \cdot mL^{-1}$	This work
	Methanol extract	*P. dactylifera* pits	IC_{50} = 6700 $\mu g \cdot mL^{-1}$	[9]
	Aqueous ammonia extracts	*Sambucus nigra* flower extract	MIC = 1500 $\mu g \cdot mL^{-1}$	[39]
		S. nigra leaf extract	MIC = 1500 $\mu g \cdot mL^{-1}$	
	Aqueous ammonia extract	*Uncaria tomentosa* bark extract	MIC = 500 $\mu g \cdot mL^{-1}$	[40]
	Aqueous extracts	*Origanum heracleoticum* inflorescences	MIC > 500,000 $\mu g \cdot mL^{-1}$	[41]
		Salvia officinalis leaves	MIC > 500,000 $\mu g \cdot mL^{-1}$	
		R. officinalis leaves and flowers	MIC > 500,000 $\mu g \cdot mL^{-1}$	
	Aqueous extracts	*Mentha piperita*	MIC >100,000 $\mu g \cdot mL^{-1}$	[42]
		Thymus vulgaris	MIC >100,000 $\mu g \cdot mL^{-1}$	
		Lavandula angustifolia	MIC >100,000 $\mu g \cdot mL^{-1}$	
	Ethyl acetate extract	*Artemisia argyi*	EC_{50} = 1227 $\mu g \cdot mL^{-1}$	[43]
	Methanol extract	*Haplophyllum tuberculatum* aerial parts	MIC > 1000 $\mu g \cdot mL^{-1}$	[44]
	Chloroform extract	*Chrysanthemum coronarium* aerial parts	MIC = 1000 $\mu g \cdot mL^{-1}$	
	Acetone extracts	*A. sativum* leaves and stems	MIC > 5000 $\mu g \cdot mL^{-1}$	[45]
		Allium sp. leaves and stems	MIC > 5000 $\mu g \cdot mL^{-1}$	
		Atropa belladonna leaves and stems	MIC > 5000 $\mu g \cdot mL^{-1}$	
		A. indica leaves and stems	MIC > 5000 $\mu g \cdot mL^{-1}$	
		Castanea sativa leaves and stems	MIC > 5000 $\mu g \cdot mL^{-1}$	
		Citrus aurantium leaves and stems	MIC > 5000 $\mu g \cdot mL^{-1}$	
		Citrus sp. leaves and stems	MIC = 5000 $\mu g \cdot mL^{-1}$	
		Inula viscosa leaves and stems	MIC > 5000 $\mu g \cdot mL^{-1}$	
		Juniperus communis leaves and stems	MIC > 5000 $\mu g \cdot mL^{-1}$	
		Olea europaea leaves and stems	MIC > 5000 $\mu g \cdot mL^{-1}$	
		Papaver rhoeas leaves and stems	MIC > 5000 $\mu g \cdot mL^{-1}$	
		Pistacia lentiscus leaves and stems	MIC > 5000 $\mu g \cdot mL^{-1}$	
		S. officinalis leaves and stems	MIC > 5000 $\mu g \cdot mL^{-1}$	
		S. nigra leaves and stems	MIC > 5000 $\mu g \cdot mL^{-1}$	

		Thymus sp. leaves and stems	MIC ≥ 5000 μg·mL^{-1}	
R. solani	Water:methanol (1:1 *v/v*)	*H. stoechas* inflorescences	MIC = 187.5 μg·mL^{-1}	This work
	Aqueous, lanolin or cocoa butter extracts	*Larrea tridentata* leaves	IC_{50} = 185–5710 μg·mL^{-1}	[46]
		F. cernua leaves	IC_{50} = 420–2120 μg·mL^{-1}	
		Agave lechuguilla leaves	IC_{50} = n.a.–17,000 μg·mL^{-1}	
		Opuntia sp. stalks	IC_{50} = 3830–20,800 μg·mL^{-1}	
		Yucca sp. leaves	IC_{50} = 8140–57,400 μg·mL^{-1}	
	Ethanol/water extracts at 4%	*Cinnamomum verum*	IR = 95.8%	[47]
		Pimpinella anisum	IR = 90.8%	
		Nigella sativa	IR = 90.5%	
		Syzygium aromaticum	IR = 100%	
	Aqueous extracts at 5, 10, and 20%	*P. hysterophorus* leaves and flowers	IR = 19.6–32.2%	[29]
		M. charantia leaves	IR = 37.7–61.1%	
		A. sativum cloves	IR = 62.2–63.3%	
		E. globulus leaves	IR = 59.6–70.7%	
		C. procera leaves	IR = 11.1%	
		A. vera leaves	IR = 33.3–38.8%	
		B. vulgaris root	IR = 36.3–40.3%	
		D. stramonium leaves	IR = 55.5–72.2%	
	Aqueous extracts at 100%	*Lawsonia inermis* leaves	IR = 100%	[48]
		Pelargonium graveolens leaves	IR = 22%	
		P. longifolia leaves	IR = 26%	
		Piper betel leaves	IR = 100%	
	Ethyl acetate, methanol or n-hexane extracts at 1000 μg·mL^{-1}	*L. inermis* leaves	IR = n.a.–100%	
		P. graveolens leaves	IR = n.a.–100%	
		P. longifolia leaves	IR = n.a.–100%	
		P. betel leaves	IR = n.a.–100%	
	Aqueous extracts at 5%	*Capsicum frutescens* fruits	IR = 71.1%	[49]*
		E. globulus leaves	IR = 37.8%	
		Eruca sativa seeds	IR = 34.4%	
		A. sativum bulbs	IR = 40%	
		Trigonella foenum-graecum seeds	IR = 42.2%	
		L. camara flowers and leaves	IR = 43.3%	
		Cymbopgon citratus leaves	IR = 55.6%	
		A. cepa bulbs or seeds	IR = 32.3–58.9%	

	S. officinalis leaves	IR = 33.3%	
Aqueous extracts at 20%	*Polystichum squarrosum* leaves	IR = 24.35%	[50]
	Adiantum venustum leaves	IR = 10.57%	
	P. hysterophorus leaves	IR = 33.12%	
	Urtica dioica leaves	IR = 18.80%	
	Cannabis sativa leaves	IR = 47.59%	
Ethanol extracts at 10,000 μg·mL^{-1}	*H. anthelmintica* fruits	IR = 100%	[25]
	C. magna fruits	IR = 17.41%	
	C. sappan barks	n.a.	
	X. lanceatum fruits	IR = 57.41%	
	C. brachiata barks	IR = 4.81%	
Aqueous or ethanol extracts at 1:1 (*w/v*)	*Eucalyptus*	IR = 32.9%	[51]*
	Pelargonium cucullatum	IR = 48.1%	
	Mentha suaveolens	IR = 35%	
	Alpinia zerumbet	IR = 53.6%	
	Artemisia capillaris	IR = 63.7%	
	R. officinalis	IR = 67.7%	
	S. aromaticum	IR = 100%	
	A. indica	IR = 38.1–87.5%	
Aqueous extracts at 15%	*A. indica*	IR = 76.67%	[52]
	Ocimum basalicum	IR = 61.11%	
	Cymbopogan flexousus	IR = 94.44%	
	T. vulgaris	IR = 94.44%	
	A. vera	IR = 61.11%	
	Tagetes patula	IR = 92.59%	
	Cordia curassavica	IR = 85.18%	
	Aegle marmelos	IR = 64.82%	
	Allium fistulosum	n.a.	
	S. aromaticum	IR = 94.44%	
	Calotropis gigantean	IR = 9.26%	
Aqueous extracts at 10%	*A. sativum* cloves	IR = 100%	[53]
	Tagetes spp. leaves	IR = 76.30%	
	E. globulus leaves	IR = 75.56%	
	C. longa rhizomes	IR = 78.52%	
	Z. officinale rhizomes	IR = 80%	

		A. cepa bulbs	IR = 37.78%	
		Ocimum spp. leaves	IR = 37.78%	
	Aqueous or ethanol extracts at 2500 μg·mL^{-1}	*Piper guinensis* leaves	IR = 84.4–100%	[54]
		O. sanctum leaves	IR = 53.8–67.1%	
		Ocimum gratissimum leaves	IR = 58.7–72.3%	
		Cymbopogon citratus leaves	IR = 72.5–86.8%	
		C. lemon leaves	IR = 9.5–20.8%	
	Chloroform, hexane or methanol extracts at 400 μg·mL^{-1}	*T. arjuna* leaves	IR = 19.81–54.07%	[26]
		C. infortunatum leaves	IR = 24.07–100%	
		A. graveolens seeds	IR = 61.11–85.93%	
		Z. officinale rhizomes	IR = 20.37–100%	
		A. sativum bulbs	IR = 20.74–27.41%	
		L. camara leaves	IR = 15.19–24.44%	
		N. tabacum leaves	IR = 26.30–37.78%	
		P. hydropiper leaves	IR = 35.56–52.96%	
		P. pinnata seeds	IR = 17.41–22.96%	
		P. longifolia leaves	IR = 16.85–100%	
		T. grandis leaves	IR = 20.74–34.81%	
	Ethanolic extract	*M. spicata* leaves and stems	MIC = 5%	[37]
S. sclerotiorum	Water:methanol (1:1 *v/v*)	*H. stoechas* inflorescences	MIC = 187.5 μg·mL^{-1}	This work
	Methanol/water extract (1:1 *v/v*)	*A. maritima* flowers	MIC = 375 μg·mL^{-1}	[28]
	Chloroform, ethyl acetate, methanol or hexane extracts	*C. nocturnum* flowers	MIC = 250–1000 μg·mL^{-1}	[31]
	Aqueous extracts	*Trachystemon orientalis* leaves or flowers	MIC = 1–7%	[55]
	Crude extracts	*R. officinalis* leaves	MIC = 10%	[56]
		Salvia fructicosa leaves	MIC = 20%	
	Ethanol extract	*M. spicata* leaves and stems	MIC = 5%	[37]
	Aqueous extract	*A. sativum* leaves	MIC = 5000 μg·mL^{-1}	[38]

* In these studies, only the most active extracts have been included in the comparison table. IR: inhibition rate; MIC: minimum inhibitory concentration; IC_{50}: half-maximal inhibitory concentration; n.a.: no activity at the highest concentration tested; n.s.: not specified.

References (reference numbers do not match those that appear in the main document)

1. Kantwa, S.; Tetarwal, J.; Shekhawat, K. In vitro effect of fungicides and phyto-extracts against *Alternaria alternata* causing leaf blight of groundnut. *IOSR Journal of Agriculture and Veterinary Science* **2014**, *7*, 28-31.
2. Singh, P.; Srivastava, D. Biofungicidal or biocontrol activity of *Lantana camara* against phytopathogenic *Alternaria alternata*. *International Journal of Pharmaceutical Sciences and Research* **2012**, *3*, 4818-4821, doi:10.13040/IJPSR.0975-8232.3(12).4818-21.
3. Carvalho, D.D.C.; Alves, E.; Camargos, R.B.; Oliveira, D.F.; Scolforo, J.R.S.; de Carvalho, D.A.; Batista, T.R.S. Plant extracts to control *Alternaria alternata* in Murcott tangor fruits. *Revista Iberoamericana de Micología* **2011**, *28*, 173-178.
4. Nagaraju, K.; Mishra, J.P.; Prasad, R.; Sekhar, J.C.; Reddy, V.P.; Kumar, S. Isolation and in vitro evaluation of different botanicals on mycelia growth of *Alternaria alternata* (Fr.) Keissler causing leaf spot of brinjal. *J. Pharmacogn. Phytochem.* **2020**, *9*, 889-891.
5. Sharma, R.L.; Ahir, R.; Yadav, S.L.; Sharma, P.; Ghasolia, R. Effect of nutrients and plant extracts on Alternaria blight of tomato caused by *Alternaria Alternata*. *Journal of Plant Diseases and Protection* **2021**, *128*, 951-960.
6. Carmello, C.R.; Magri, M.M.R.; Cardoso, J.C. Cinnamon extract and sodium hypochlorite in the in vitro control of *Fusarium oxysporum* f. sp. *lycopersici* and *Alternaria alternata* from tomato. *Journal of Phytopathology* **2022**, *170*, 802-810.
7. Shingne, A.W.; Giri, G.; Bagade, A.R. In vitro evaluation of fungicides, botanicals and bio-agents against *Alternaria alternata* causing leaf spot disease of niger. *International Journal of Chemical Studies* **2020**, *8*, 3360-3364.
8. Rizwana, H.; Bokahri, N.A.; Alsahli, S.A.; Al Showiman, A.S.; Alzahrani, R.M.; Aldehaish, H.A. Postharvest disease management of *Alternaria* spots on tomato fruit by *Annona muricata* fruit extracts. *Saudi J. Biol. Sci.* **2021**, *28*, 2236-2244.
9. Bokhari, N.A.; Perveen, K. In vitro inhibition potential of *Phoenix dactylifera* L. extracts on the growth of pathogenic fungi. *Journal of Medicinal Plants Research* **2012**, *6*, 1083-1088.
10. Rodino, S.; Buţu, M.; Petrache, P.; Butu, A.; Cornea, C.P. Antifungal activity of four plants against *Alternaria alternata*. *Scientific Bulletin. Series F. Biotechnologies* **2014**, *18*, 60-65.
11. Chaudhary, S.; Singh, H. In-vitro evaluation of different botanicals against *Alternaria alternata* causing Alternaria leaf spot of ber (*Zizyphus mauritiana* Lamk.). *International Journal of Economic Plants* **2021**, *8*, 40-44.
12. Kharbadkar, V.P.; Kadam, J.; Dalvi, N.; Phondekar, U.; Chaure, N.; Gaonkar, R.; Joshi, M. In vitro exploration of botanicals and fungicides against *Alternaria alternata* inciting leaf blight disease of chrysanthemum. *Pharma Innovation Journal* **2022**, *11*, 68-71.
13. Teixeira, A.; Sánchez-Hernández, E.; Noversa, J.; Cunha, A.; Cortez, I.; Marques, G.; Martín-Ramos, P.; Oliveira, R. Antifungal activity of plant waste extracts against phytopathogenic fungi: *Allium sativum* peels extract as a promising product targeting the fungal plasma membrane and cell wall. *Horticulturae* **2023**, *9*, 136, doi:10.3390/horticulturae9020136.
14. Ogbebor, N.; Adekunle, A.; Enobakhare, D. Inhibition of *Colletotrichum gloeosporioides* (Penz) Sac. causal organism of rubber (*Hevea brasiliensis* Muell. Arg.) leaf spot using plant extracts. *African Journal of Biotechnology* **2007**, *6*, 213-218.
15. Necha, L.L.B.; Luna, L.B.; Torres, K.B.; Baños, S.B. Antifungal activity of leaf and stem extracts from various plant species on the incidence of *Colletotrichum gloeosporioides* of papaya and mango fruit after storage. *Revista Mexicana de Fitopatología* **2002**, *20*, 8-12.
16. Gwa, V.; Nwankiti, A. Efficacy of some plant extracts in in vitro control of *Colletotrichum* species, causal agent of yam (*Dioscorea rotundata* Poir) tuber rot. *Asian Journal of Plant Science and Research* **2017**, *7*, 8-16.
17. Peraza-Sánchez, S.R.; Chan-Che, E.O.; Ruiz-Sánchez, E. Screening of Yucatecan plant extracts to control *Colletotrichum gloeosporioides* and isolation of a new pimarene from *Acacia pennatula*. *J. Agric. Food. Chem.* **2005**, *53*, 2429-2432.
18. Shovan, L.; Bhuiyan, M.; Begum, J.; Pervez, Z. In vitro control of *Colletotrichum dematium* causing anthracnose of soybean by fungicides, plant extracts and *Trichoderma harzianum*. *Int. J. Sustain. Crop Prod* **2008**, *3*, 10-17.
19. Deressa, T.; Lemessa, F.; Wakjira, M. Antifungal activity of some invasive alien plant leaf extracts against mango (*Mangifera indica*) anthracnose caused by *Colletotrichum gloeosporioides*. *International Journal of Pest Management* **2015**, *61*, 99-105.
20. López-Velázquez, J.G.; Delgado-Vargas, F.; Ayón-Reyna, L.E.; López-Angulo, G.; Bautista-Baños, S.; Uriarte-Gastelum, Y.G.; López-López, M.E.; Vega-García, M.O. Postharvest application of

partitioned plant extracts from Sinaloa, Mexico for controlling papaya pathogenic fungus *Colletotrichum gloeosporioides*. *Journal of Plant Pathology* **2021**, *103*, 831-842.
21. Masangwa, J.; Aveling, T.; Kritzinger, Q. Screening of plant extracts for antifungal activities against Colletotrichum species of common bean (*Phaseolus vulgaris* L.) and cowpea (*Vigna unguiculata* (L.) Walp). *The Journal of Agricultural Science* **2013**, *151*, 482-491.
22. Hernández-Ceja, A.; Loeza-Lara, P.; Espinosa-García, F.; García-Rodríguez, Y.; Medina-Medrano, J.; Gutiérrez-Hernández, G.; Ceja-Torres, L. In vitro antifungal activity of plant extracts on pathogenic fungi of blueberry. *Plants* **2021**, *10*, 852, doi:10.3390/plants10050852.
23. Mukherjee, A.; Khandker, S.; Islam, M.; Shahid, S.B. Efficacy of some plant extracts on the mycelial growth of *Colletotrichum gloeosporioides*. *Journal of the Bangladesh Agricultural University* **2011**, *9*, 43-47, doi:10.3329/jbau.v9i1.8742.
24. Silva, A.V.; Yerena, L.R.; Necha, L.L.B. Chemical profile and antifungal activity of plant extracts on *Colletotrichum* spp. isolated from fruits of *Pimenta dioica* (L.) Merr. *Pestic. Biochem. Physiol.* **2021**, *179*, 104949.
25. Jantasorn, A.; Moungsrimuangdee, B.; Dethoup, T. In vitro antifungal activity evaluation of five plant extracts against five plant pathogenic fungi causing rice and economic crop diseases. *Journal of Biopesticides* **2016**, *9*, 1.
26. Choudhury, D.; Anand, Y.R.; Kundu, S.; Nath, R.; Kole, R.K.; Saha, J. Effect of plant extracts against sheath blight of rice caused by *Rhizoctonia solani*. *J. Pharmacogn. Phytochem.* **2017**, *6*, 399-404.
27. Rashid, T.S.; Awla, H.K.; Sijam, K. Antifungal effects of *Rhus coriaria* L. fruit extracts against tomato anthracnose caused by *Colletotrichum acutatum*. *Ind Crops Prod* **2018**, *113*, 391-397, doi:10.1016/j.indcrop.2018.01.066.
28. Sánchez-Hernández, E.; Martín-Ramos, P.; Navas Gracia, L.M.; Martín-Gil, J.; Garcés-Claver, A.; Flores-León, A.; González-García, V. *Armeria maritima* (Mill.) Willd. flower hydromethanolic extract for cucurbitaceae fungal diseases control. *Molecules* **2023**, *28*, 3730, doi:10.3390/molecules28093730.
29. Cherkupally, R.; Kota, S.R.; Amballa, H.; Reddy, B.N. In vitro antifungal potential of plant extracts against *Fusarium oxysporum*, *Rhizoctonia solani* and *Macrophomina phaseolina*. *Annals of Plant Sciences* **2017**, *6*, 1676, doi:10.21746/aps.2017.9.2.
30. Rongai, D.; Pulcini, P.; Pesce, B.; Milano, F. Antifungal activity of pomegranate peel extract against fusarium wilt of tomato. *European Journal of Plant Pathology* **2017**, *147*, 229-238.
31. Al-Reza, S.M.; Rahman, A.; Ahmed, Y.; Kang, S.C. Inhibition of plant pathogens in vitro and in vivo with essential oil and organic extracts of *Cestrum nocturnum* L. *Pestic. Biochem. Physiol.* **2010**, *96*, 86-92, doi:10.1016/j.pestbp.2009.09.005.
32. Bashar, M.; Chakma, M. In vitro control of *Fusarium solani* and *F. oxysporum* the causative agent of brinjal wilt. *Dhaka University Journal of Biological Sciences* **2014**, *23*, 53-60.
33. De Rodriguez, D.J.; Hernández-Castillo, D.; Angulo-Sánchez, J.; Rodríguez-García, R.; Quintanilla, J.V.; Lira-Saldivar, R. Antifungal activity in vitro of *Flourensia* spp. extracts on *Alternaria* sp., *Rhizoctonia solani*, and *Fusarium oxysporum*. *Ind Crops Prod* **2007**, *25*, 111-116.
34. El-Mohamedy, R.S.; Abdalla, A.M. Evaluation of antifungal activity of *Moringa oleifera* extracts as natural fungicide against some plant pathogenic fungi in vitro. *Journal of Agricultural Technology* **2014**, *10*, 963-982.
35. Satish, S.; Raghavendra, M.; Raveesha, K. Antifungal potentiality of some plant extracts against *Fusarium* sp. *Archives of Phytopathology and Plant Protection* **2009**, *42*, 618-625.
36. Nelson, D.; Beattie, K.; McCollum, G.; Martin, T.; Sharma, S.; Rao, J.R. Performance of natural antagonists and commercial microbiocides towards in vitro suppression of flower bed soil-borne *Fusarium oxysporum*. *Advances in Microbiology* **2014**, *4*, 151-159, doi:10.4236/aim.2014.43020.
37. Naji, E.T. Study of alkaloids, phenols and terpenes of *Mentha spicata* as a fungicide against *Rhizoctonia solani*, *Sclerotinia sclerotiorum* and *Fusarium oxysporum*. *International Journal of Advanced Biological Research* **2017**, *7*, 345-354.
38. Singh, U.; Pathak, K.; Khare, M.; Singh, R. Effect of leaf extract of garlic on *Fusarium oxysporum* f. sp. *ciceri*, *Sclerotinia sclerotiorum* and on gram seeds. *Mycologia* **1979**, *71*, 556-564.
39. Sánchez-Hernández, E.; Balduque-Gil, J.; González-García, V.; Barriuso-Vargas, J.J.; Casanova-Gascón, J.; Martín-Gil, J.; Martín-Ramos, P. Phytochemical profiling of *Sambucus nigra* L. flower and leaf extracts and their antimicrobial potential against almond tree pathogens. *Int. J. Mol. Sci.* **2023**, *24*, 1154, doi:10.3390/ijms24021154.
40. Sánchez-Hernández, E.; Martín-Ramos, P.; Martín-Gil, J.; Santiago-Aliste, A.; Hernández-Navarro, S.; Oliveira, R.; González-García, V. Bark extract of *Uncaria tomentosa* L. for the control of strawberry phytopathogens. *Horticulturae* **2022**, *8*, doi:10.3390/horticulturae8080672.

41. Salamone, A.; Zizzo, G.; Scarito, G. The antimicrobial activity of water extracts from Labiatae. *Acta Hort* **2006**, *723*, 465-470, doi:10.17660/ActaHortic.2006.723.67.
42. Erdoğan, O.; Celik, A.; Zeybek, A. In vitro antifungal activity of mint, thyme, lavender extracts and essential oils on *Verticillium dahliae* Kleb. *Fresenius Environmental Bulletin* **2016**, *25*, 4856-4862
43. Wang, Y.; Li, J.; Chen, Q.; Zhou, J.; Xu, J.; Zhao, T.; Huang, B.; Miao, Y.; Liu, D. The role of antifungal activity of ethyl acetate extract from *Artemisia argyi* on *Verticillium dahliae*. *Journal of Applied Microbiology* **2022**, *132*, 1343-1356.
44. Abdelgaleil, S.; Saad, M.; Ariefta, N.; Shiono, Y. Antimicrobial and phytotoxic activities of secondary metabolites from *Haplophyllum tuberculatum* and *Chrysanthemum coronarium*. *S. Afr. J. Bot.* **2020**, *128*, 35-41.
45. Varo, A.; Mulero-Aparicio, A.; Adem, M.; Roca, L.; Raya-Ortega, M.; López-Escudero, F.; Trapero, A. Screening water extracts and essential oils from Mediterranean plants against *Verticillium dahliae* in olive. *Crop Protection* **2017**, *92*, 168-175.
46. Castillo, F.; Hernández, D.; Gallegos, G.; Mendez, M.; Rodríguez, R.; Reyes, A.; Aguilar, C.N. In vitro antifungal activity of plant extracts obtained with alternative organic solvents against *Rhizoctonia solani* Kühn. *Ind Crops Prod* **2010**, *32*, 324-328, doi:10.1016/j.indcrop.2010.05.013.
47. Al-Askar, A.A.; Rashad, Y.M. Efficacy of some plant extracts against *Rhizoctonia solani* on pea. *Journal of Plant Protection Research* **2010**, *50*, 239-243, doi:10.2478/v10045-010-0042-0.
48. Seema, M.; Sreenivas, S.; Rekha, N.; Devaki, N. In vitro studies of some plant extracts against *Rhizoctonia solani* Kuhn infecting FCV tobacco in Karnataka Light Soil, Karnataka, India. *Journal of Agricultural technology* **2011**, *7*, 1321-1329.
49. Abd-El-Khair, H.; El-Gamal Nadia, G. Effects of aqueous extracts of some plant species against *Fusarium solani* and *Rhizoctonia solani* in *Phaseolus vulgaris* plants. *Archives of Phytopathology and Plant Protection* **2011**, *44*, 1-16.
50. Tapwal, A.; Garg, S.; Gautam, N.; Kumar, R. In vitro antifungal potency of plant extracts against five phytopathogens. *Brazilian Archives of Biology and Technology* **2011**, *54*, 1093-1098.
51. San Aye, S.; Matsumoto, M. Effect of some plant extracts on *Rhizoctonia* spp. and *Sclerotium hydrophilum*. *J. Med. Plants Res* **2011**, *5*, 3751-3757.
52. Persaud, R.; Khan, A.; Isaac, W.-A.; Ganpat, W.; Saravanakumar, D. Plant extracts, bioagents and new generation fungicides in the control of rice sheath blight in Guyana. *Crop Protection* **2019**, *119*, 30-37, doi:10.1016/j.cropro.2019.01.008.
53. Chakrapani, K.; Sinha, B.; Chanu, W.T.; Chakma, T.; Siram, T. Assessing in vitro antifungal activity of plant extracts against *Rhizoctonia solani* causing sheath blight of rice (*Oryza sativa* L). *J. Pharmacogn. Phytochem.* **2020**, *9*, 1497-1501.
54. Amadioha, A. Fungicidal activity of some plant extracts against *Rhizoctonia*. *Archives of Phytopathology & Plant Protection* **2001**, *33*, 509-517.
55. Onaran, A.; Yılar, M. Antifungal and herbicidal activity of Trachystemon orientalis (L.) G. Don against some plant pathogenic fungi and Cuscuta campestris Yunck. *Iğdır Univ. J. Inst. Sci. & Tech.* **2018**, *8*, 37-43.
56. Goussous, S.; Mas' ad, I.; Abu El-Samen, F.; Tahhan, R. In vitro inhibitory effects of rosemary and sage extracts on mycelial growth and sclerotial formation and germination of *Sclerotinia sclerotiorum*. *Archives of Phytopathology and Plant Protection* **2013**, *46*, 890-902.
